# Individual brain metabolic connectome indicator based on Kullback-Leibler Divergence Similarity Estimation predicts progression from mild cognitive impairment to Alzheimer’s dementia

**DOI:** 10.1007/s00259-020-04814-x

**Published:** 2020-04-22

**Authors:** Min Wang, Jiehui Jiang, Zhuangzhi Yan, Ian Alberts, Jingjie Ge, Huiwei Zhang, Chuantao Zuo, Jintai Yu, Axel Rominger, Kuangyu Shi, Alzheimer’s Disease Neuroimaging Initiative

**Affiliations:** 1 Shanghai Institute for Advanced Communication and Data Science, Shanghai University, 99 Shangda Road, Shanghai, 200444 China; 2 Key laboratory of Specialty Fiber Optics and Optical Access Networks, Joint International Research Laboratory of Specialty Fiber Optics and Advanced Communication, Shanghai University, Shanghai, China; 3 Department of Nuclear Medicine, Inselspital, University Hospital Bern, Bern, Switzerland; 4 Department of Nuclear Medicine, PET Center, Huashan Hospital, Fudan University, 518 Wuzhong Dong Road, Shanghai, 201103 China; 5 Institute of Functional and Molecular Medical Imaging, Fudan University, Shanghai, China; 6 Department of Neurology, Huashan Hospital, Fudan University, Shanghai, China; 7 Department of Informatics, Technische Universität München, Munich, Germany

**Keywords:** Alzheimer’s disease, Mild cognitive impairment, Connectome, FDG PET, Conversion prediction

## Abstract

**Purpose:**

Positron emission tomography (PET) with ^18^F-fluorodeoxyglucose (FDG) reveals altered cerebral metabolism in individuals with mild cognitive impairment (MCI) and Alzheimer’s dementia (AD). Previous metabolic connectome analyses derive from groups of patients but do not support the prediction of an individual’s risk of conversion from present MCI to AD. We now present an individual metabolic connectome method, namely the Kullback-Leibler Divergence Similarity Estimation (KLSE), to characterize brain-wide metabolic networks that predict an individual’s risk of conversion from MCI to AD.

**Methods:**

FDG-PET data consisting of 50 healthy controls, 332 patients with stable MCI, 178 MCI patients progressing to AD, and 50 AD patients were recruited from ADNI database. Each individual’s metabolic brain network was ascertained using the KLSE method. We compared intra- and intergroup similarity and difference between the KLSE matrix and group-level matrix, and then evaluated the network stability and inter-individual variation of KLSE. The multivariate Cox proportional hazards model and Harrell’s concordance index (C-index) were employed to assess the prediction performance of KLSE and other clinical characteristics.

**Results:**

The KLSE method captures more pathological connectivity in the parietal and temporal lobes relative to the typical group-level method, and yields detailed individual information, while possessing greater stability of network organization (within-group similarity coefficient, 0.789 for sMCI and 0.731 for pMCI). Metabolic connectome expression was a superior predictor of conversion than were other clinical assessments (hazard ratio (HR) = 3.55; 95% CI, 2.77–4.55; *P* < 0.001). The predictive performance improved further upon combining clinical variables in the Cox model, i.e., C-indices 0.728 (clinical), 0.730 (group-level pattern model), 0.750 (imaging connectome), and 0.794 (the combined model).

**Conclusion:**

The KLSE indicator identifies abnormal brain networks predicting an individual’s risk of conversion from MCI to AD, thus potentially constituting a clinically applicable imaging biomarker.

**Electronic supplementary material:**

The online version of this article (10.1007/s00259-020-04814-x) contains supplementary material, which is available to authorized users.

## Introduction

With currently some 50 million cases worldwide, Alzheimer’s disease (AD) is the most common cause of dementia, and its prevalence is projected to grow rapidly over the next decades [1]. Mild cognitive impairment (MCI) is a preclinical stage of AD, in which individuals are free from overt cognitive and behavioral symptoms, but are showing subtle prodromal signs of dementia [2]. A subgroup of MCI individuals has especially high risks for imminent progression to AD, while the remainder may undergo no further cognitive decline in the coming years. Given the unpredictable course of MCI, it is important to develop sensitive biomarkers and predictors of an individual’s risk of progression from MCI to AD.

Various recent studies show that biomarkers derived from ^18^F-fluorodeoxyglucose positron emission tomography (FDG-PET) can accurately predict conversion from MCI to AD [3–5]. In particular, machine learning and deep learning tools have successfully classified different prodromal stages in this conversion. An FDG-PET study using multi-scale deep convolutional neural network analysis to predict future cognitive decline in MCI patients, attained a predictive accuracy of 84.2% [6]. Applying an optimized semi-quantitative FDG-PET method, Pagani et al. correctly identified 89% of MCI patients who converted to AD within 5 years of follow-up [7]. Notably, Blazhenets et al. proposed a novel method with spatial covariance mapping of the FDG-PET signal to identify an AD conversion-related pattern of cerebral metabolism [8]. These methods emphasize regional FDG uptake or quantitative characteristics of metabolically abnormal regions in PET images, without fully considering the metabolic interactions of between-regions, thus potentially losing relevant information related to concerning individual differences in metabolic topology.

In contrast to earlier work on group-based metabolic patterns, brain network analysis based on graph theory could offer an individualized assessment of metabolic patterns predictive of conversion. Brain network science has already contributed to obtain a better understanding of the pathophysiology of AD [9]. Furthermore, it has delineated local and global brain metabolic abnormalities typical for MCI and AD, while affording an effective diagnostic biomarker to identify AD at an early phase [10, 11]. Recently, a series of novel analytic methodologies have been proposed to investigate metabolic networks in PET imaging [12, 13]. The metabolic connections in FDG-PET images are typically estimated via regional differences in standard uptake value (SUV), which is a surrogate index of the cerebral metabolic rate for glucose. However, most of the metabolic connectome studies have hitherto adopted group-level network methods for metabolic network modeling. For example, some FDG-PET studies derive functional metabolic connectivity networks using sparse inverse covariance estimation [13], while other studies implemented the Pearson’s correlation to obtain group-level metabolic networks [12, 14]. While successful in revealing network abnormalities in clinical groups, we point out that such group-level metabolic network analyses sacrifice critical individual-level information.

Inspired by MR-based structural studies using the Kullback-Leibler divergence similarity estimation (KLSE) [15–17], we now apply the concept of relative entropy to develop a new analytic methodology for individual-level metabolic brain network construction in FDG-PET imaging. By FDG-PET images from the Alzheimer’s Disease Neuroimaging Initiative (ADNI) database, we test the KLSE method for predicting the conversion of MCI to AD as assessed with clinical follow-up. We firstly establish the validity and efficacy of the relative entropy concept to construct a metabolic network for each subject and secondly use the KLSE indicators to predict the risk of conversion in MCI patients during a 3-year follow-up.

## Methods

### Kullback-Leibler divergence similarity estimation method

The KLSE method has been successfully implemented with structural MRI data for individual morphological brain network analysis [15–17], but has not yet been used for constructing individual metabolic networks from FDG-PET imaging. We suppose that the FDG-PET signal across brain regions indicates metabolic connections subserving inter-regional information transfer. The relatively high resting signal-to-noise FDG-PET signal in a volume of interest (VOI) reflects the relative glucose metabolism rate, i.e., energy consumption demand of this region. Regional metabolism indexed by local FDG radiotracer uptake is an index of afferent synaptic activity [18–20]. This putative relationship offers a plausible approach to characterize inter-neuronal information transfer. Statistical relationships of the similarity of cerebral glucose metabolism in any two regions depicted through KLSE (the relative entropy) can then delineate individual metabolic connections, as summarized in Fig. A1.

For globally normalized FDG uptake maps, the intensity of voxels within each of *n* specific VOIs are extracted and used to estimate the probability density function (PDF) of this VOI using non-parametric kernel density estimation.

We next derived the metabolic connections as the symmetric Kullback-Leibler (KL) divergence (relative entropy), according to the mathematical equation:

1 $$ {D}_{\mathrm{KL}}\left(P\Big\Vert Q\right)=\underset{X}{\int}\left(P(x)\log \frac{P(x)}{Q(x)}+Q(x)\log \frac{Q(x)}{P(x)}\right) dx $$

where *P* and *Q* represent the probability density functions (PDFs) of voxel intensities in a pair of VOIs. Finally, we calculated the metabolic connectivity strength of pairwise VOIs by KL divergence as follows:

2 $$ \mathrm{KLS}\Big(P\mid \left|Q\right)={e}^{-{D}_{\mathrm{KL}}\left(P\Big\Vert Q\right)} $$

As a measure of metabolic connectivity, we obtained an adjacency matrix from the KLSE. This adjacency matrix describes pairwise metabolic connectivity, where each *ij*^th^ element of this matrix denotes the metabolic connection strength between region *i* and *j*.

### Materials

**Fig. 1:**
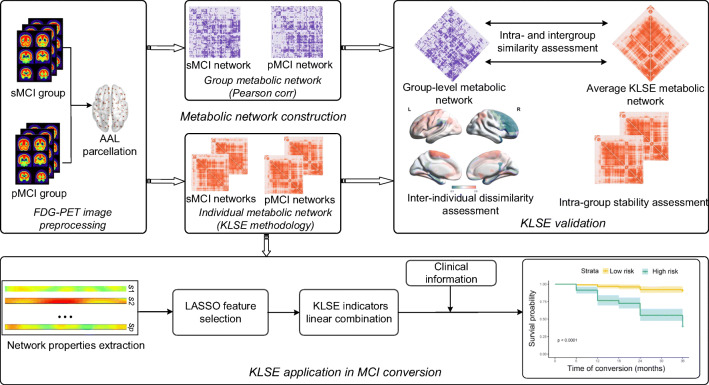
The flowchart of experimental procedures in this study

To validate the effectiveness of KLSE in individual metabolic connectome networks and to test its applicability in predicting the risk of MCI conversion, we conducted a series of experiments, as summarized in Fig. 1. After constructing individual metabolic networks via our KLSE method, we compared intra- and intergroup similarity and difference between the KLSE and group-level matrices by using Pearson’s correlation. Then, we applied KLSE individual metabolic network analysis to characterize MCI-conversion indicators, thus defining an image-derived biomarker to predict the individual risk of conversion from MCI to AD.

#### Subjects

The data used in this study were obtained from the Alzheimer’s Disease Neuroimaging Initiative (ADNI) database (adni.loni.usc.edu) and its extensions. The primary goal of ADNI has been to test whether serial MRI, PET, other biological markers, and clinical and neuropsychological assessment can be combined to predict and measure the progression of MCI and early AD. The institutional review boards of ADNI provided review and approval of the ADNI data collection protocol. Written, informed consent had been obtained from each subject.

We investigated two independent cohorts of subjects with baseline FDG-PET images collected from the ADNI databases. Our cohort A contained 510 subjects with either stable MCI (sMCI, *n* = 332) or progressive MCI (pMCI, *n* = 178) with clinical follow-up; we used the FDG-PET images from this cohort to established predictive modeling and to test the validity of our models. Our cohort B consisted of 50 AD patients and 50 age/gender-matched healthy controls; we used the FDG-PET images from this cohort to evaluate the diagnostic utility of our predictive models.

Detailed eligibility criteria for these participants are shown in Fig. A2. In brief, eligible participants with MCI underwent FDG-PET scanning and clinical cognitive evaluations at baseline and were clinically followed-up during at least 36 months. These MCI participants were stratified post hoc as (1) stable MCI subjects with baseline MCI diagnosis (including early and late MCI) who had not converted to AD at follow-up, and (2) progressive MCI patients who had converted to AD within the follow-up interval. All cases with ultimate AD diagnosis satisfied the diagnostic criteria according to the National Institute of Neurological and Communicative Disorders and Stroke and Alzheimer’s disease and Related Disorders Association [21]. Equal numbers of cohort A subjects were randomly assigned to training or test datasets using a computer-generated randomization list. The demographic and clinical characteristics of all participants are summarized in Table 1. The clinical characteristics did not significantly differ between training and test datasets for the sMCI or pMCI subgroups (*P* > 0.05). There were significant differences in age, MMSE score, and APOE ε4 positivity between the sMCI and pMCI groups, but no between training and test datasets.

**Table 1:** Clinical and baseline demographic characteristics of all participants

Cohort	Group		Sex (M/F)	Age (years)	Education (years)	MMSE score	APOE ε4 positive rate
Cohort A	Training dataset (*n* = 255)	sMCI (*n* = 166)	90/76	71.2 ± 7.81	16.1 ± 2.62	28.4 ± 1.51	45.2%
		pMCI (*n* = 89)	52/37	73.2 ± 7.56	16.4 ± 2.47	26.9 ± 1.54	64.1%
		*P* value	0.52^a^	0.045^b^	0.36^c^	< 0.001^b^	0.0041^a^
	Test dataset (*n* = 255)	sMCI (*n* = 166)	85/81	70.6 ± 6.81	16.1 ± 2.64	28.4 ± 1.53	34.3%
		pMCI (*n* = 89)	50/39	74.1 ± 6.21	15.5 ± 2.79	27.1 ± 2.03	73.0%
		*P* value	0.45^a^	0.01^b^	0.11^c^	<0.001^b^	<0.001^a^
Cohort B	HC (*n* = 50)		24/26	74.6 ± 3.17	15.8 ± 2.53	29.1 ± 1.01	28%
	AD (*n* = 50)		28/22	74.8 ± 2.75	15.7 ± 2.64	23.1 ± 2.23	74%
	*P* value		0.55^a^	0.71^b^	0.87^c^	< 0.001^b^	< 0.001^a^

*P*^a^, the chi-square test; *P*^b^, the two-sample *t* test; *P*^c^, the Wilcoxon rank-sum test. *sMCI*, stable MCI; *pMCI*, progressive MCI; *MMSE*, mini-mental state examination; APOE ε4 positive rate, positive or negative for the presence of at least one ε4 allele

Data are given as mean ± SD

#### FDG-PET images acquisition and preprocessing

Baseline FDG-PET images in a state of rest were acquired 30–35 min after administration of 185 ± 18.5 MBq FDG, with acquisition details as outlined in the study protocols of the ADNI database. The images were spatially normalized to a PET template in Montreal Neurological Institute (MNI) brain space and smoothened with a Gaussian filter of 8 mm full-width at half-maximum (FWHM). Individual PET images were intensity normalized to the global mean brain uptake and automatically parcellated into 90 regions of interest (ROIs) defined by the automated anatomical labeling (AAL) atlas. All preprocessing was performed using Statistical Parametric Mapping software (SPM12; Wellcome Department of Imaging Neuroscience, Institute of Neurology, London, UK) implemented in MATLAB 2016b (MathWorks Inc., Sherborn, MA).

### KLSE metabolic network construction

For depicting the individual metabolic network, we represent brain nodes by 90 telencephalic VOIs from the AAL atlas parcellation. Globally normalized FDG uptake in each VOI was used to generate a region × region correlation matrix (90 × 90) for each subject. We applied the KLSE method to individual FDG-PET image to construct a correlation matrix which included pairwise regional metabolic connections. PET images of all participants underwent individual metabolic network construction for further analysis. Figure A3 shows an individual metabolic network topography of one representative sMCI subject (male; age, 72 years; MMSE, 30; APOE4, negative) and one pMCI subject (male; age, 70 years; MMSE, 27; APOE4, positive). Using the KLSE method, significant individual differences in these two brain network connectomes are conspicuous.

### KLSE validation

In this step, we implemented three evaluation experiments to characterize the validity and effectiveness of KLSE.

#### KLSE validity assessment

To examine the validity of KLSE for metabolic network construction, we compared results of KLSE with those of a conventional group-level network estimation method by characterizing pathological changes in the metabolic connection patterns. We calculated the MCI-conversion patterns from the individual- and group-level network seen in cohort A. The group-level network was constructed using Pearson’s correlation as described elsewhere [12, 14]. We obtained the metabolic difference patterns associated with conversion from MCI to AD. In addition, we quantified and compared the number of pathologically altered metabolic connectivity within brain regions in MCI, respectively.

#### KLSE stability assessment

To further evaluate the network stability of KLSE, we performed an inter-subject similarity analysis by defining two subgroups from cohort A, each consisting of 20 sMCI subjects or 20 pMCI subjects, who had otherwise similar demographic characteristics. The similarity between the 20 metabolic networks derived from these 20 sMCI or pMCI subjects was measured by averaging the correlation coefficients between any pair of networks as follows:

3 $$ R=E\left[\mathrm{corr}\left(F\left({s}_p\right),F\left({s}_q\right)\right)\right],p,q\in 1,2,\cdots 20;\kern0.5em p\ne q $$

where *F*(*s*_*p*_) represents the vector of all metabolic connections in subject *p*, corr indicates the Spearman rank correlation, and *E* indicates the expected value or average value.

#### KLSE inter-individual dissimilarity

Cortical metabolic connection patterns vary between individuals. Therefore, to determine the efficiency of KLSE in describing this heterogeneity, we explored inter-individual variations in metabolic networks of each brain region using a previously proposed measure of dissimilarity [22, 23]. For a given brain region *i*, the inter-individual dissimilarity between the metabolic networks derived from MCI patients is estimated from the following formula:

4 $$ Vi=E\left[1-\mathrm{corr}\left({F}_i\left({s}_p\right),{F}_i\left({s}_q\right)\right)\right],\kern0.5em p\ne q $$

where *F*_*i*_(*s*_*p*_) is a 1×90 vector of metabolic connectivities between region *i* and the other regions in MCI subject *s*, and corr represents the Spearman rank-correlation. We applied Z-normalization to the spatial maps of inter-individual dissimilarity to further compare the regional dissimilarity map.

### KLSE application in MCI conversion

#### KLSE indicators extraction and selection

For the metabolic network of each subject, we calculated 457 network properties, of which seven are global network properties and 450 are regional network properties. The seven global properties are clustering coefficient, characteristic path length (L), small-worldness (S), global efficiency, transitivity, assortativity coefficient, and modularity. We also examined the regional network properties for a given node of the 90 VOIs for five other properties: betweenness centrality, degree, local efficiency, vulnerability, and local clustering coefficient. The details of these calculations are provided in Table A1. These network properties were computed using the Brain Connectivity Toolbox.

To identify the network properties associated with conversion from MCI to AD, we applied an L1-penalized logistic regression model based on the least absolute shrinkage and selection operator (LASSO) to evaluate the contribution of individual properties in the training dataset (cohort A). Metabolic connectome expression (MCE), obtained by the linear combination of the top network properties, provides information about the interaction between VOIs and the specific metabolic abnormalities associated with the conversion to AD. We used the test dataset (cohort A) to evaluate the performance of the logistic regression model based connectome method. The relative FDG uptake value in an AD-meta-ROI was also implemented to compare the diagnostic performance of our proposed connectome method [7]. Furthermore, we tested the connectome diagnostic performance in cohort B, consisting of 50 AD patients and 50 age/gender-matched healthy controls.

#### Cox model analysis

To consider different times to conversion among pMCI subjects and to evaluate more strictly the metabolic connectome expression as a predictive biomarker, we applied Cox proportional hazard regression analysis to the clinical variables (MMSE score, APOE ε4 genotype) and the metabolic connectome expression derived from the training dataset in cohort A. For each pMCI subject, we registered survival time as the interval between the time of baseline PET imaging and the time of initial AD diagnosis (or the last follow-up time for sMCI subjects). The Z-statistic for each continuous covariate was used to calculate a hazard ratio (HR) of the risk for conversion as a function of the number of standard deviations of an increase in the covariates. We then performed four multivariable Cox model analyses with age and sex as factors, including the following variables: (1) clinical (MMSE, APOE ε4), (2) metabolic connectome expression (MCE), (3) group-level pattern expression score (PES) [8], and (4) combination of all variables (MMSE, APOE ε4, MCE). To assess the model’s validity, each Cox model was applied independently to the test dataset.

We statistically evaluated the predictive performance of the Cox model in terms of Harrell’s concordance index (C-index). To evaluate the validity of a given Cox model, we applied the Cox model to the test dataset and obtained the prognostic index (PI) for each subject. To compare the hazard ratio (HR) of each independent predictor variables and to test for confounding variables also contributing to the model, we calculated a Cox model including all indicators in the training dataset. We also performed a Kaplan-Meier analysis of overall survival to conversion using the PI values as a stratification variable, with equally sized risk groups (low-risk and high-risk) based on the ranked PI values.

### Statistical analysis

Clinical and demographic characteristics were compared between groups using a two-sample *t* test, the chi-square test, or the Wilcoxon rank-sum test. Hazard ratios and associated 95% confidence intervals (CIs) were evaluated using the Cox proportional hazards regression model with Efron’s method. Between-group differences in Kaplan-Meier overall survival for MCI patients were evaluated using a log-rank test, stratified as described above. All analyses were considered significant for *P* < 0.05 (2-tailed). The statistical analyses of conversion-free survival were performed using R (version 3.61) employing the survival and glmnet packages.

## Results

### KLSE validation

#### KLSE validity assessment

**Fig. 2:**
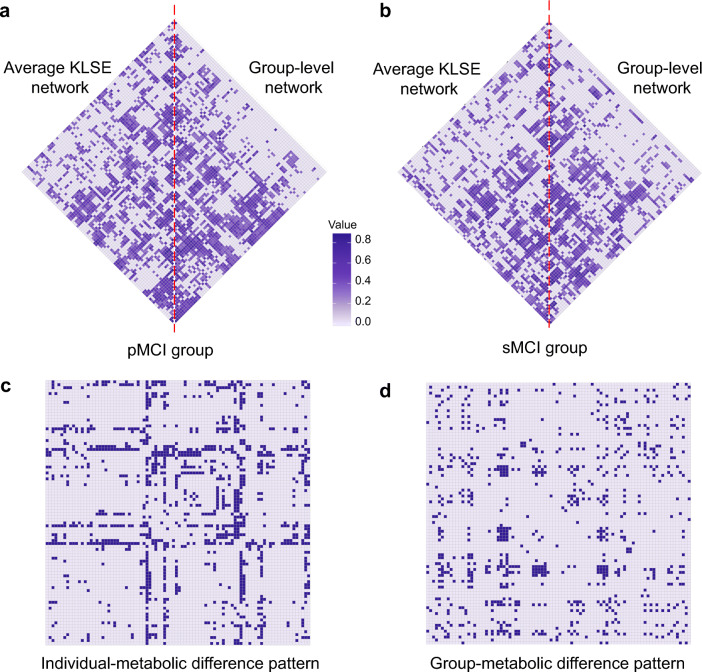
Metabolic network topology in the pMCI group (**a**) and sMCI group (**b**). The matrices represent the mean average metabolic network based on the KLSE method (left triangle) and a group-level metabolic network based on a conventional Pearson’s correlation method (right triangle). The color intensity indicates the strength of metabolism correlations. Metabolic difference patterns of predictive of MCI-conversion are shown for individual- (**c**) and group-level (**d**) networks. For calculating the individual-level pattern, we first applied Fisher’s Z-transformation to the metabolic network of each MCI subject. Next, we compared the Z-coefficients of the pMCI and sMCI groups using a two-sample *t* test with false discovery rate (FDR) correction. For calculating the corresponding group-level pattern, we likewise applied Fisher’s Z-transformation and corrected *P* values for FDR. *P* < 0.05 was considered significant. Each row (column) in the matrix corresponds to one of the 90 VOIs. Purple cells represent significantly different connectivity

We constructed a metabolic brain network from each subject’s FDG-PET image using the KLSE method. There is compelling similarity between individual- and group-level network topographies in the pMCI group (Fig. 2a) and the sMCI group (Fig. 2b). Metabolic patterns predictive of MCI-conversion at the individual and group levels are shown in Fig. 2c, d. These figures show that metabolic connectivity in the pMCI group differs significantly from that in the sMCI group (*P* < 0.05, FDR corrected). For individual-level metabolic patterns, we found pathologically altered of patterns predictive of 5% for the frontal connectivity, 13.7% for occipital connectivity, 26.4% for parietal connectivity and 13.9% for temporal lobe connectivity. In the group-level metabolic patterns, corresponding values were 17.7%, 13.6%, 10.9%, and 6.6%, respectively. The pattern difference shows that our proposed individual network method captures more than twice as many changes in parietal and temporal lobes that are predictive of conversion to AD relative to the typical group-level method. Moreover, the novel application of the KLSE method discovered a large number of abnormal metabolic connectivity findings predictive of conversion in the precuneus, inferior temporal, and posterior cingulate, this is in comparison to the group-level analysis.

#### KLSE stability assessment

We explored the inter-subject network similarity in the sMCI or pMCI groups. Here, the within-group similarity coefficient of metabolic networks was 0.789 in the sMCI group, versus 0.731 in the pMCI group, indicating lower individual network variability among those who progressed during follow-up, suggesting that our proposed method has considerable stability for detecting metabolic network architectures.

#### KLSE inter-individual dissimilarity

**Fig. 3:**
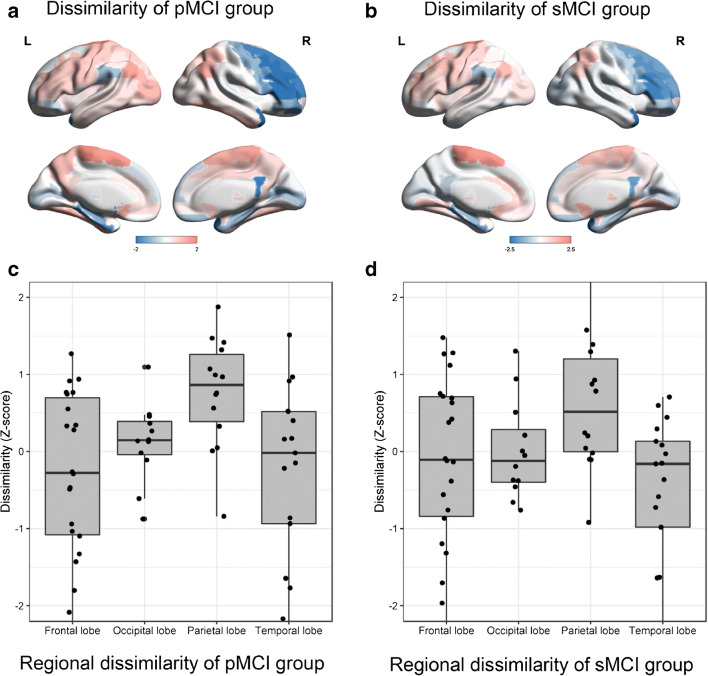
Box and whisker plots of inter-individual dissimilarity of metabolic networks across pMCI patients (*N* = 178) (**a**), and across sMCI patients (*N* = 332) (**b**). Inter-individual dissimilarity is higher for parietal lobe than other lobes in the pMCI group (**c**) (all *P* < 0.01) and the sMCI (**d**) group (all *P* < 0.05)

As shown in Fig. 3, we found that inter-individual dissimilarity was higher in paracentral, angular, and olfactory regions in the pMCI group, whereas dissimilar regions included the paracentral, thalamic, and lingual regions in the sMCI group. The mean inter-individual dissimilarity in parietal lobe was higher than in the other lobes across the group of 178 pMCI patients (all *P* < 0.01; Z-score, 0.766 for parietal, 0.123 for occipital, − 0.303 for frontal and − 0.308 for temporal lobes). Similar to findings in the pMCI group, the sMCI group showed higher inter-individual dissimilarity in the parietal lobe than in the other lobes (all *P* < 0.05; Z-scores, 0.616, 0.008, − 0.121 and − 0.528, respectively). These findings underline that our individual-level network construction methodology may detect more individual variation in the metabolic network organization, thus capturing more idiosyncratic or individual details.

### KLSE methodology application in MCI conversion

Using the multivariate logistic regression method, we identified 13 local network properties associated with conversion from MCI to AD. These properties were linearly combined in the logistic regression model. However, this model did not yield a significant between-group difference in all global properties (*P* > 0.05).

Average network properties for the sMCI and pMCI groups in the training and test datasets showed significant between-group differences (Fig. A4; *P* < 0.01). The network property of vulnerability was significantly higher in pMCI subjects in the precuneus, lingual, temporoparietal cortex, and precentral gyrus relative to that in the sMCI subjects (*P* < 0.05). The local efficiencies in inferior temporal gyrus and inferior frontal cortex were significantly lower in the pMCI group relative to sMCI subjects, as was the property of betweenness centrality in putamen (*P* < 0.05). There were no significant differences in the other network properties between the sMCI and pMCI groups (*P* > 0.05).

**Fig. 4:**
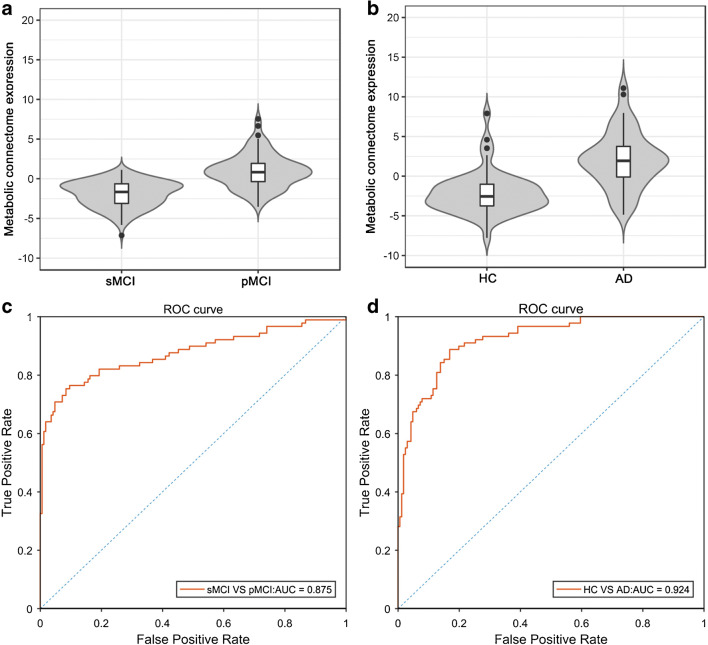
The expression scores of metabolic connectome model (MCE) was increased in pMCI groups compared with sMCI patients in test dataset (**a**), and also higher in AD patients compared with healthy people (**b**). ROC curve for metabolic connectome expression in cohort A, i.e., progressive versus stable MCI (**c**) and cohort B, i.e., healthy controls versus Alzheimer’s disease (**d**)

The MCE model differed significantly between sMCI and pMCI groups with an AUC of 0.875 in the test dataset (Fig. 4a and c), which is superior to conventional FDG uptake marker (AUC, 0.721). The metabolic connectome model gave distinct results between HC and AD groups (Fig. 4b, *P* < 0.001). As with the distinction between sMCI and pMCI groups, the ROC analysis for the mixed HC/AD group once more showed that MCE had excellent predictive performance (Fig. 4d, AUC = 0.924).

### Cox proportional hazards analysis

**Fig. 5:**
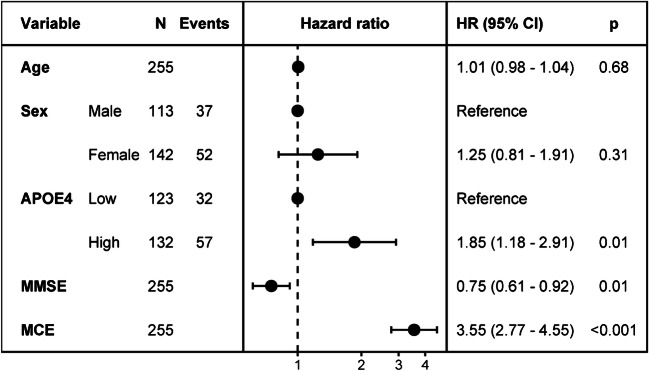
Hazard ratios of different predictors

**Fig. 6:**
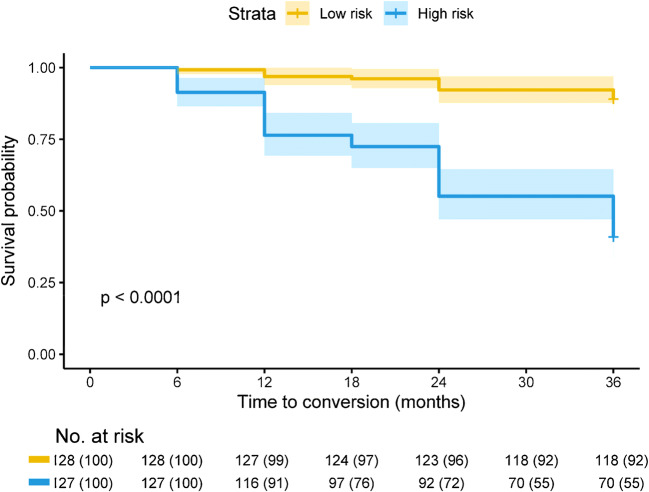
Kaplan-Meier of overall survival in test dataset of cohort A for the combined model

As summarized in Fig. 5, MCE, MMSE, and APOE ε4 status all proved to be significant predictors for conversion from MCI to AD, with MCE emerging as the most significant factor (HR, 3.55; 95% CI, 2.77–4.55; *P* < 0.001). Among the four Cox models in Table A3, the performance of the imaging connectome model revealed that MCE was a significant independent predictor for conversion (HR, 3.80; 95% CI, 2.99–4.82; *P* < 0.001) rather than traditional group-level model (pattern model; pattern expression score-PES: HR, 3.18; 95% CI, 2.56–3.9). Age and sex were no significant variables in clinical or combined models (*P* > 0.1). The imaging connectome model had superior predictability over the clinical model in the test dataset (C-index, 0.750 vs 0.728). As expected, the combined model also had superior predictive performance in the test dataset (C-index, 0.794). Overall survival to conversion was significantly prolonged in the low-risk group compared to the high-risk group (HR, 9.074; 95% CI, 4.93–16.7; *P* < 0.001) (Fig. 6).

## Discussion

We present an individual-level metabolic network construct approach for FDG-PET imaging and apply it to the task of predicting individual risk of conversion from MCI to AD. Recapitulating previous studies about MCI conversion prediction, these works indeed have successfully differentiated prodromal stages in MCI conversion. Nevertheless, such approaches cannot depict individual pathophysiology details and downstream clinical therapeutic strategies, whereas the ascendancy of our novel KLSE approach can afford individual risk estimation. The symptoms of many neurological and psychiatric diseases are mappable to specific functional networks of interconnected brain regions.

Hitherto, all FDG-PET imaging studies of metabolic networks have used group-level analyses, which potentially sacrifice or obscure salient individual differences within a group. The KLSE approach has proven successful for individual structural MR-based analyses [16, 17], but has not yet been applied to metabolic maps. Based on a calculation of relative entropy, the KLSE can quantify the inter-regional metabolic interactions for the construction of an individual’s metabolic brain network. The putative association of FDG metabolism with afferent synaptic activity implies that elements of the connectome are metabolically coupled [20, 24]. The intra-regional similarities calculated from KLSE in the present case of FDG-PET are a surrogate measure of the metabolic connectivity between brain regions. In making a series of evaluations of FDG-PET data from the extended ADNI dataset, we evaluated the KLSE method’s validity and effectiveness for predicting conversion of MCI to AD, as compared with a traditional group-level method based on Pearson’s correlations. We found that the individualized metabolic KLSE network analysis revealed subtle deviations in metabolic connectivity that were powerfully predictive of conversion of MCI to AD. Indeed, the KLSE method outperformed the traditional group-level approach for revealing patterns of altered metabolic connectivity predictive of conversion. Our findings of salient connectivity patterns in the parietal and occipital lobes, recapitulate results of previous FDG-PET group-based comparisons [8, 25]. In addition, we found that KLSE has considerable intra-group stability for resolving metabolic network organization, and highlights an especially pronounced inter-individual dissimilarity of metabolic connectivity of the parietal lobe of the pMCI group (Fig. 3). Overall, we find that applying KLSE to FDG-PET data is a compelling approach for revealing metabolic connectivity networks, having superior performance relative to a traditional group-level method, and revealing novel insights into the nature of metabolic disturbances in the progression from MCI to AD.

In our analysis, we exploited a priori knowledge about the clinical trajectory of 510 MCI patients in the ADNI database to search for predictive patterns in the baseline FDG-PET images. In so doing, we discriminated metabolic connectivity patterns between sMCI and pMCI subgroups with roughly similar baseline cognitive deficits. We explored the abnormal metabolic network metrics associated with subsequent AD conversion and used a logistic regression approach to develop a metabolic connectome biomarker. We next validated this biomarker relative to conventional clinical characteristics and known risk factors using Cox proportional hazard regression models. Our composite model sensitively discriminated sMCI and pMCI patients (AUC 0.827 for the test dataset) from their baseline FDG-PET images, thus predicting their risk of AD conversion during a 3-year follow-up. Notably, there was a highly significant negative correlation between MCE and clinical MMSE score for the composite population (*r* = − 0.483, *P* < 0.001), indicating a strong link between connectome expression and cognitive function (Fig. A5).

Because our present brain connectome approach can measure local network properties and the entire network, it could powerfully identify salient properties predictive of conversion from MCI to AD. In this regard, our main finding was that conversion to AD entails disruption of modules (or subnetworks) of the global network architecture and a loss of connectivity between those modules. A number of previous PET studies have likewise revealed an early failure of brain modules in relation to the onset of cognitive dysfunction [26]. In the present work, we observed lower local efficiency in pMCI compared to sMCI patients, particularly in inferior temporal gyrus and inferior frontal cortex and higher vulnerability in precuneus cortex, lingual, precentral gyrus, and temporoparietal cortex. With the progression of MCI, these affected brain regions fail to metabolically compensate in those MCI subjects destined to undergo further cognitive decline. This latter result is consistent with previous studies showing the failure of network components early in the neurodegeneration process leading to AD [27]. Furthermore, we tested for analogous changes of connectome properties in the contrast between HC and AD patients, as summarized in Fig. A6. As expected, the sMCI and pMCI groups occupied a position in connectome topology intermediate to the HC and AD groups. Our cross-sectional FDG PET data imply a progressive loss of connectivity in the metabolic network on the trajectory to AD. Network properties in metabolic connectivity in precuneus, middle frontal region, and temporal pole appeared to change progressively in our cross-sectional comparison of HC, sMCI, pMCI, and AD groups. The results are in accord with previous MRI studies showing analogous network changes in MCI conversion [27, 28].

Among the limitations of this study, we note that the endpoint of semi-quantitative FDG-PET is not entirely specific to neuronal metabolism, but rather can reflect non-specific aspects of the progressive neurodegeneration progression [29]. The implementation of KLSE method of this work used for FDG PET images without partial volume effect (PVE) correction. However, PVE correction can be used for metabolic network construction, and the results agreed (Table A4). Our interpretation of a link between metabolic network failure and AD pathology remains to be confirmed by multimodal imaging with tracers such as amyloid-β or tau deposition. Therefore, future studies may require data other than the glucose metabolism employed in the current study to fully verify the applicability of metabolic connectome method.

## Conclusion

This study presents an advanced connectome analysis of FDG-PET images based on a novel application of KLSE entropy measures, not previously applied to the task of metabolic connectome analysis. This method sheds new light on the network abnormality underlying the risk for conversion from MCI to AD. Importantly, we present a novel prognostic score for conversion risk that is additive to clinical risk stratification procedures, attaining a C-index as high as 0.794 for the combined model. By providing remarkably quantitative biomarkers in individual subjects, the connectome model along with clinical and metabolic pattern characteristics provides a more comprehensive method to determining the risk in MCI subjects to convert to AD in the coming years.

## Electronic supplementary material

ESM 1 (DOCX 19685 kb).
