# Supplementary material for: Individual brain metabolic connectome indicator based on Kullback-Leibler Divergence Similarity Estimation predicts progression from mild cognitive impairment to Alzheimer’s dementia
Source: Eur J Nucl Med Mol Imaging. 2020 Apr 22;47(12):2753–64. doi: 10.1007/s00259-020-04814-x (PMC7567735; doi:10.1007/s00259-020-04814-x)
Supplement: Supplementary file 1 — (DOCX 19685 kb). [file 259_2020_4814_MOESM1_ESM.docx]

**Supplemental appendix**

***Kullback-Leibler divergence similarity estimation (KLSE) method considerations***

Statistical relationships of the similarity of cerebral glucose metabolism in any two regions depicted through KLSE (the relative entropy) delineate individual metabolic connections. For globally normalized FDG uptake intensity of voxels within each of *n* specific VOIs are extracted such as left angular or left fusiform regions in **Figure A1**, and used to estimate the probability density function (PDF) of this VOI using non-parametric kernel density estimation (KDE), in which kernel width is estimated by the solve-the-equation bandwidth approach [1]. Here, it is plausible to quantify the glucose metabolic relations between brain regions with the PDFs of their regional tracer metabolism. The characteristic function was calculated as follows:

|  | $\hat{\varphi}\left( t \right)=\frac{1}{n}\sum_{j=1}^{n} e^{itx_{j}}$ | (1) |
| --- | --- | --- |

where $x_{j}$ represents an array quantifying the metabolic intensity of each voxel within the VOI. To circumvent the problem of diverging integral we used the Gaussian function as a damping function:

|  | $\psi\left( t \right)=e^{-\pi t^{2}}$ | (2) |
| --- | --- | --- |

Next, we apply the Fourier transform formula and derive the density estimation using the following equation:

|  | $\hat{f}\left( x \right)=\frac{1}{2\pi}\int_{-\infty}^{+\infty} \hat{\varphi}\left( t \right)\psi_{h}\left( t \right)e^{-itx}dt=\frac{1}{2\pi}\int_{-\infty}^{+\infty} \frac{1}{n}\sum_{j=1}^{n} e^{it{(x}_{j}-x)}\psi\left( ht \right)dt=\frac{1}{nh}\sum_{j=1}^{n} \frac{1}{2\pi}\int_{-\infty}^{+\infty} e^{-i\left( ht \right)\frac{x-x_{j}}{h}}\psi\left( ht \right)d(ht)=\frac{1}{nh}\sum_{j=1}^{n} K(\frac{x-x_{j}}{h})$ | (3) |
| --- | --- | --- |

where *K* is the Fourier transform of the damping function. The PDFs of left angular and left fusiform were estimated as **Figure A1**.

KL divergence is a natural method for estimating the dissimilarity/similarity between two probability distributions. In this work, a similarity measurement based on the symmetric KL divergence was employed to quantify the similarity of two PDFs of local brain regions. We next derived the metabolic connectivity strength via the symmetric Kullback-Leibler (KL) divergence (relative entropy), according to the mathematical equation:

|  | $D_{KL}\left( P\vert\vert Q \right)= \int_{X} (P\left( x \right)log\frac{P\left( x \right)}{Q\left( x \right)}+Q(x)log\frac{Q(x)}{P(x)})dx$ | (4) |
| --- | --- | --- |

where *P* and *Q* represent the probability density functions (PDFs) of voxel intensity in a pair of VOIs, and *X* represent a set which spans defined intervals of *P* and *Q*. More generally, the range of integral is from $-\infty$ to $+\infty$.

Finally, we calculated the similarity of pairwise VOIs by KL divergence as follows:

|  | $KLS(P\vert\left\vert Q \right)=e^{-D_{KL}(P\vert\vert Q)}$ | (5) |
| --- | --- | --- |

The KLS ranges from 0 to 1, where 1 is for two identical distributions. As a result, the metabolic connectivity strength between left angular and left fusiform is 0.37 in **Figure A1**.

As a measure of metabolic connectivity, we obtained an adjacency matrix from the KLSE. This adjacency matrix describes pairwise metabolic connectivity, where each *ij* th element of this matrix denotes the metabolic connection strength between region *i* and *j*.

Understanding the limitations of KLSE method is critical to employing this approach to estimating metabolic connectivity. The same spatial resolution of post-processed images is recommended and it will reduce the impact of different scanning devices. To avoid any bias that might be introduced by resampling peak cluster voxels, standardized regions of interest (ROIs) defined according to the AAL atlas is great choice. There are no special considerations for the uniformity of tracer uptake in the ROI. Accurate assessment will require extensive validation for FDG tracer and tissue under consideration.

Table A1. Mathematical definitions of connectome network properties.

| Network properties | Description | Weighted and undirected definitions |
| --- | --- | --- |
| Base concepts and notation | $N$ is the set of all nodes in the network, and $n$ is the number of nodes.  $L$ is the set of all links in the network, and $l$ is the number of links.  $(i,j)$ is a link between $i$ and $j (i,j\in N)$.  $w_{ij}$ is connectivity strength between *i* and *j.* $d_{ij}^{w}$ is shorted weighted path length between $i$ and $j$. $L^{w}$ is weighted characteristic path length. $E^{w}$ is weighted global efficiency.  $C^{w}$ is weighted clustering coefficient. $T^{w}$ is weighted transitivity. $Q^{w}$ is weighted modularity. $r^{w}$ is weighted assortativity coefficient. $S^{w}$ is weighted network small-worldness. $E_{loc}^{w}$ is weighted local efficiency. | Links $(i,j)$ are associated with connection weights $w_{ij}.0\leq w_{ij}\leq1$ for all $i$ and $j$.  $l^{w}$ is the sum of all weights in the network, computed as $l^{w}=\sum_{i,j\in N} w_{ij}$. |
| Degree | The number of links connected to a node. | Weighted degree of $i$, $k_{i}^{w}=\sum_{i,j\in N} w_{ij}$ |
| Shortest path length | A basis for measuring integration. | Shorted weighted path length between $i$ and $j$, $d_{ij}^{w}=\sum_{a_{uv}\in g_{i\to j}^{w}} f\left( w_{uv} \right)$,  where $f$ is a map from weight to length and $g_{i\to j}^{w}$ is the shortest weighted path between $i$ and $j$. |
| Characteristic path length | The average shortest path length in the network [2]. | Weighted characteristic path length, $L^{w}=1/n\times\sum_{i\in N} {\sum_{j\in N,j\neq i} d_{ij}^{w}}/{(n-1)}$,  $d_{ij}^{w}$ is the shorted weighted path length between *i* and *j.* |
| Global efficiency | The average inverse shortest path length in the network, and is inversely related to the characteristic path length. | Weighted global efficiency [3], $E^{w}=1/n\times\sum_{i\in N} {\sum_{j\in N,j\neq i} {{(d}_{ij}^{w})}^{-1}}/{(n-1)}$ |
| Clustering coefficient | The fraction of triangles around a node and is equivalent to the fraction of node’s neighbors that are neighbors of each other [2]. | Weighted clustering coefficient,  $C^{w}=1/n\times\sum_{i\in N} {{2t}_{i}^{w}}/{k_{i}(k_{i}-1)}$. $t_{i}^{w}=1/2{\sum_{h,j\in N} {(w}_{hj}w_{ij}w_{ih})}^{1/3}$  $t_{i}^{w}$ is weighted geometric mean of triangles around *i*. |
| Transitivity | The ratio of triangles to triplets in the network and is an alternative to the clustering coefficient. | Weighted transitivity [4]  $T^{w}=\sum_{i\in N} {{2t}_{i}^{w}}/{\sum_{i\in N} k_{i}(k_{i}-1)}$. |
| Modularity | A statistic that quantifies the degree to which the network may be subdivided into such clearly delineated groups. | Weighted modularity, $Q^{w}=1/{l^{w}}\times\sum_{i,j\in N} {[w}_{ij}-k_{i}^{w}k_{j}^{w}/l^{w}]\delta_{m_{i},m_{j}}$  where $m_{i}$ is the module containing node *i*, and $\delta_{m_{i},m_{j}}$ = 1 if $m_{i}$ = $m_{j}$, and 0 otherwise. |
| Assortativity coefficient | A correlation coefficient between the degrees of all nodes on two opposite ends of a link. | Weighted assortativity coefficient [4],$r^{w}=l^{-1}\sum_{(i,j)\in L} w_{ij}k_{i}^{w}k_{j}^{w}-\left[ l^{-1}\sum_{\left( i,j \right)\in L} 1/2w_{ij}k_{i}^{w}k_{j}^{w} \right]^{2}/l^{-1}\sum_{\left( i,j \right)\in L} 1/2w_{ij}(\left( k_{i}^{w} \right)^{2}+{{(k}_{j}^{w})}^{2})- \left[ l^{-1}\sum_{\left( i,j \right)\in L} 1/2w_{ij}{(k}_{i}^{w}+k_{j}^{w}) \right]^{2}$ |
| Small-worldness | A measure of network efficiency [5]. | Weighted network small-worldness, $S^{w}=C^{w}/C_{rand}^{w}$ / $L^{w}/L_{rand}^{w}$ |
| Local efficiency | The local efficiency is the global efficiency computed on the neighborhood of the node, and is related to the clustering coefficient. | Weighted local efficiency (L., 1979).$E_{loc}^{w}=1/n\sum_{i\in N} \sum_{j,h\in N,j\neq i} \left( w_{ij}w_{ih}\left[ d_{jh}^{w}\left( N_{i} \right)^{-1} \right] \right)^{\frac{1}{3}}/k_{i}(k_{i}-1)$ |
| Betweenness centrality | The fraction of all shortest paths in the network that contain a given node [6]. | Betweenness centrality is computed equivalently on weighted and directed networks, provided that path lengths are computed on respective weighted or directed paths. |
| Vulnerability | A measure that quantify the resilience for a given node [7]. | When remove a node the network efficiency changes. |

Table A2. The logistic regression model of metabolic connectome network.

| Variables | Coefficient | Std.Error | Wald$\chi^{2}$ | P-value (Wald) | $\hat{\mathrm{OR}}$ |
| --- | --- | --- | --- | --- | --- |
| Vulnerability of Precuneus | .616 | .243 | 6.431 | .011 | 1.852 |
| Degree of Frontal_Mid | 1.320 | .264 | 25.055 | .000 | 3.743 |
| Vulnerability of Hippocampus | .976 | .238 | 16.816 | .000 | 2.653 |
| Local efficiency of Temporal | -.815 | .223 | 13.386 | .000 | .443 |
| Vulnerability of Lingual | -1.232 | .261 | 22.323 | .000 | .292 |
| BC of Putamen | -.712 | .255 | 7.798 | .005 | .490 |
| Local efficient of Frontal_Inf_Orb | .839 | .249 | 11.340 | .001 | 2.314 |
| C of Paracentral_Lobule | -.306 | .211 | 2.097 | .148 | .737 |
| Vulnerability of Precentral | -.575 | .216 | 7.096 | .008 | .563 |
| BC of Cuneus | -.428 | .181 | 5.586 | .018 | .652 |
| Vulnerability of Temporal_Pole_Sup | .493 | .232 | 4.518 | .034 | 1.638 |
| Vulnerability of Parietal_Sup | -.318 | .228 | 1.955 | .162 | .727 |
| BC of Cingulum_Mid | .097 | .194 | .251 | .616 | 1.102 |
| Constant variable | -1.213 | .222 | 29.740 | .000 | .297 |

Note: BC: Betweenness Centrality; C: cluster efficiency.

Table A3. Cox proportional hazard model analyses for overall survival.

| Model | predictor | Hazard ratio | *P*-value | Harrell's concordance index (Training) | Harrell's concordance index (Test) | AIC |
| --- | --- | --- | --- | --- | --- | --- |
| Imaging connectome model | Age | 1.01 (0.97 – 1.03) | 0.95 | 0.858 | 0.75 | 809.7 |
|  | Sex | 1.13 (0.74 – 1.72) | 0.58 |  |  |  |
|  | MCE | 3.80 (2.99 – 4.82) | < 0.001 |  |  |  |
| Imaging group-level pattern model | Age | 0.99 (0.96 – 1.0) | 0.47 | 0.822 | 0.73 | 845.6 |
|  | Sex | 1.10 (0.61 – 1.4) | 0.64 |  |  |  |
|  | PES | 3.18 (2.56 – 3.9) | < 0.001 |  |  |  |
| Clinical model | Age | 1.01 (0.98 – 1.04) | 0.36 | 0.749 | 0.728 | 913.2 |
|  | Sex | 1.08 (0.70 – 1.66) | 0.72 |  |  |  |
|  | MMSE | 0.57 (0.47 – 0.69) | < 0.001 |  |  |  |
|  | APOE | 1.62 (1.01 - 2.58) | 0.043 |  |  |  |
| Combined model | Age | 1.01 (0.98 - 1.04) | 0.68 | 0.871 | 0.794 | 796.2 |
|  | Sex | 1.25 (0.81 - 1.91) | 0.31 |  |  |  |
|  | MMSE | 0.75 (0.61 - 0.92) | 0.01 |  |  |  |
|  | APOE | 1.85 (1.18 - 2.91) | 0.01 |  |  |  |
|  | MCE | 3.55 (2.77 – 4.55) | < 0.001 |  |  |  |

Table A4. The metabolic connectome expressions (MCE) over different post-processes (with and without PVE correction).

| No. | MCE scores (sMCI group) | | No. | MCE scores (pMCI group) | |
| --- | --- | --- | --- | --- | --- |
|  | No-PVE | PVE |  | No-PVE | PVE |
| 1 | 0.468 | 0.473 | 1 | 0.650 | 0.685 |
| 2 | 0.253 | 0.237 | 2 | 0.640 | 0.575 |
| 3 | 0.286 | 0.258 | 3 | 0.581 | 0.622 |
| 4 | 0.252 | 0.213 | 4 | 0.362 | 0.412 |
| 5 | 0.692 | 0.674 | 5 | 0.418 | 0.484 |
| 6 | 0.282 | 0.251 | 6 | 0.626 | 0.742 |
| 7 | 0.158 | 0.197 | 7 | 0.769 | 0.797 |
| 8 | 0.203 | 0.221 | 8 | 0.411 | 0.496 |
| 9 | 0.170 | 0.154 | 9 | 0.686 | 0.734 |
| 10 | 0.065 | 0.051 | 10 | 0.780 | 0.691 |
| 11 | 0.501 | 0.458 | 11 | 0.305 | 0.421 |
| 12 | 0.487 | 0.437 | 12 | 0.362 | 0.292 |
| 13 | 0.502 | 0.462 | 13 | 0.798 | 0.842 |
| 14 | 0.283 | 0.324 | 14 | 0.411 | 0.468 |
| 15 | 0.271 | 0.186 | 15 | 0.748 | 0.753 |
| 16 | 0.395 | 0.356 | 16 | 0.801 | 0.846 |
| 17 | 0.141 | 0.123 | 17 | 0.155 | 0.186 |
| 18 | 0.260 | 0.216 | 18 | 0.438 | 0.323 |
| 19 | 0.178 | 0.194 | 19 | 0.542 | 0.694 |
| 20 | 0.571 | 0.519 | 20 | 0.998 | 0.962 |
| 21 | 0.119 | 0.094 | 21 | 0.984 | 0.989 |
| 22 | 0.375 | 0.347 | 22 | 0.510 | 0.613 |
| 23 | 0.149 | 0.129 | 23 | 0.759 | 0.763 |
| 24 | 0.218 | 0.264 | 24 | 0.173 | 0.213 |
| 25 | 0.313 | 0.287 | 25 | 0.555 | 0.607 |
| 26 | 0.224 | 0.259 | 26 | 0.794 | 0.819 |
| 27 | 0.145 | 0.127 | 27 | 0.686 | 0.725 |
| 28 | 0.431 | 0.356 | 28 | 0.632 | 0.668 |
| 29 | 0.200 | 0.204 | 29 | 0.452 | 0.411 |
| 30 | 0.533 | 0.591 | 30 | 0.974 | 0.981 |
| *P*-value | 0.699 | | *P*-value | 0.635 | |
| *ICC* | 0.97, 95% CI: 0.93-0.986 | | *ICC* | 0.95, 95% CI: 0.897-0.979 | |
| *Variance* | 9.1% | | *Variance* | 7.58% | |

Note: The images both FDG-PET and MRI scans were collected at the baseline visit (age/gender-match), including 30 sMCI (age: 72.2±3.63 years; sex: 15/15) and 30 pMCI patients (age: 72.4±4.17 years; sex: 15/15).

ICC: Intraclass correlation coefficient. Variance: within-subject coefficient of variance.


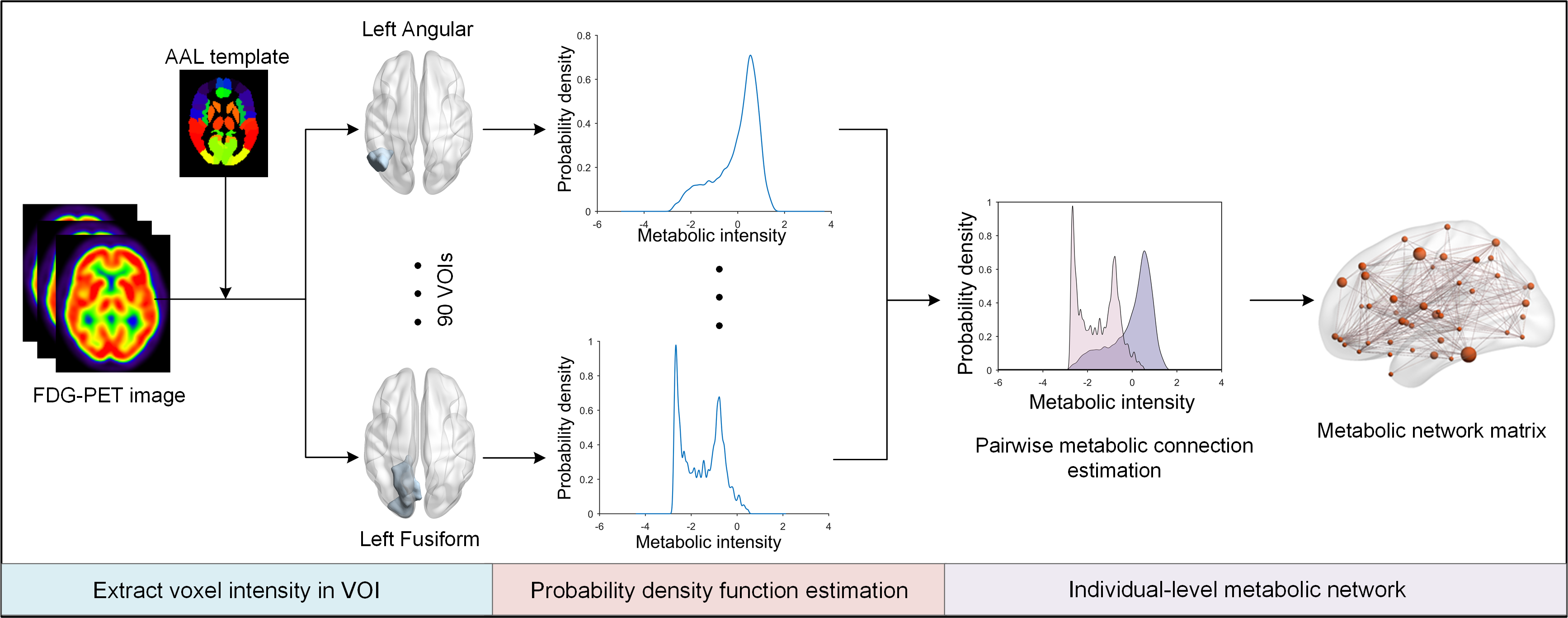


**Fig. A1** Illustration of Kullback-Leibler Divergence Similarity Estimation (KLSE) procedure as applied for maps of cerebral metabolism. A globally normalized individual FDG-PET image is automatically parcellated into volumes of interest (VOIs) based on anatomic templates in the AAL atlas. The set of voxel uptake values within each VOI is plotted as a probability density function (PDF). Next, pairwise metabolic connections are derived via the KLSE method, and finally a metabolic network matrix is constructed for each individual


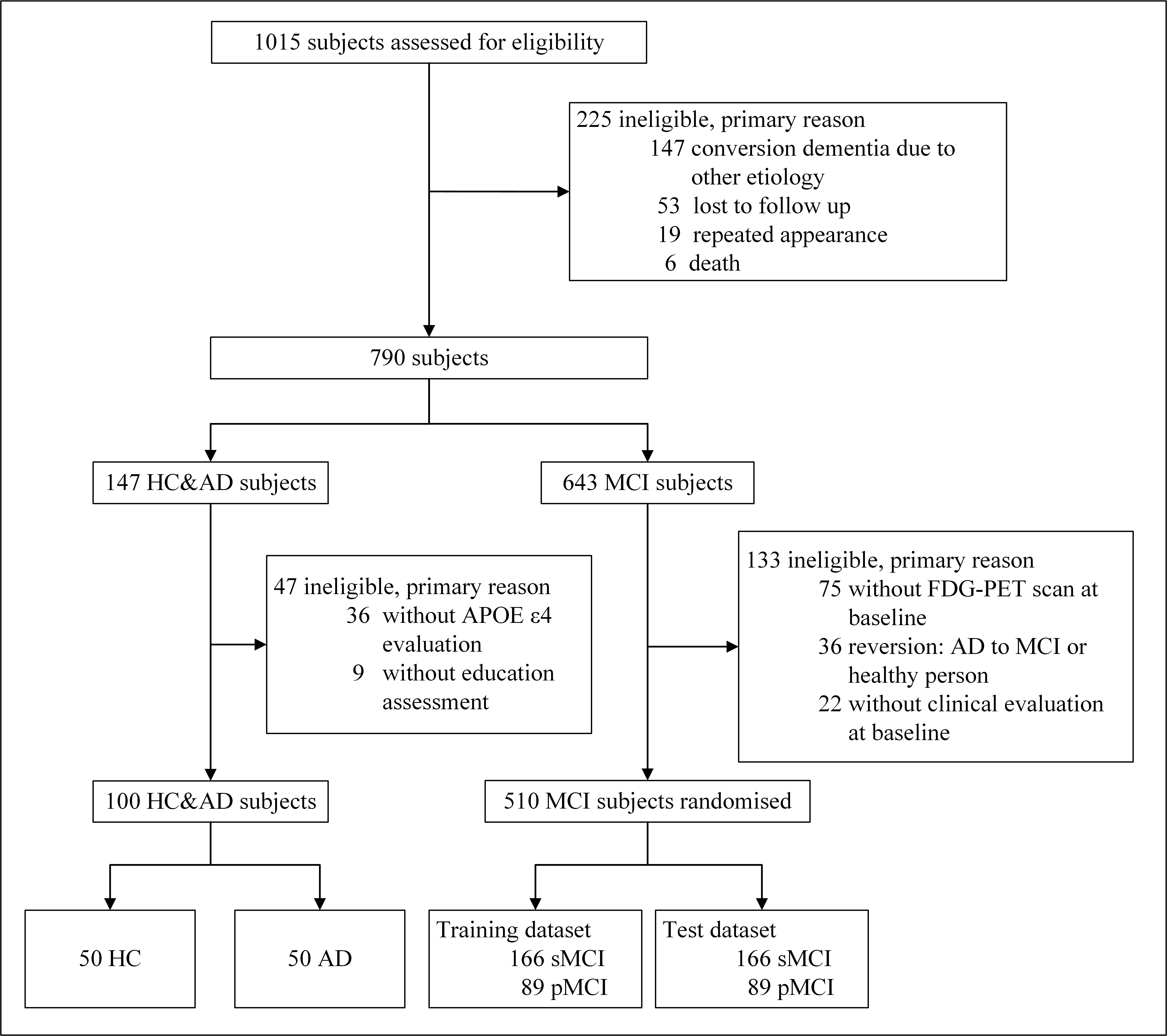


**Fig. A2** Trial profile of all subjects


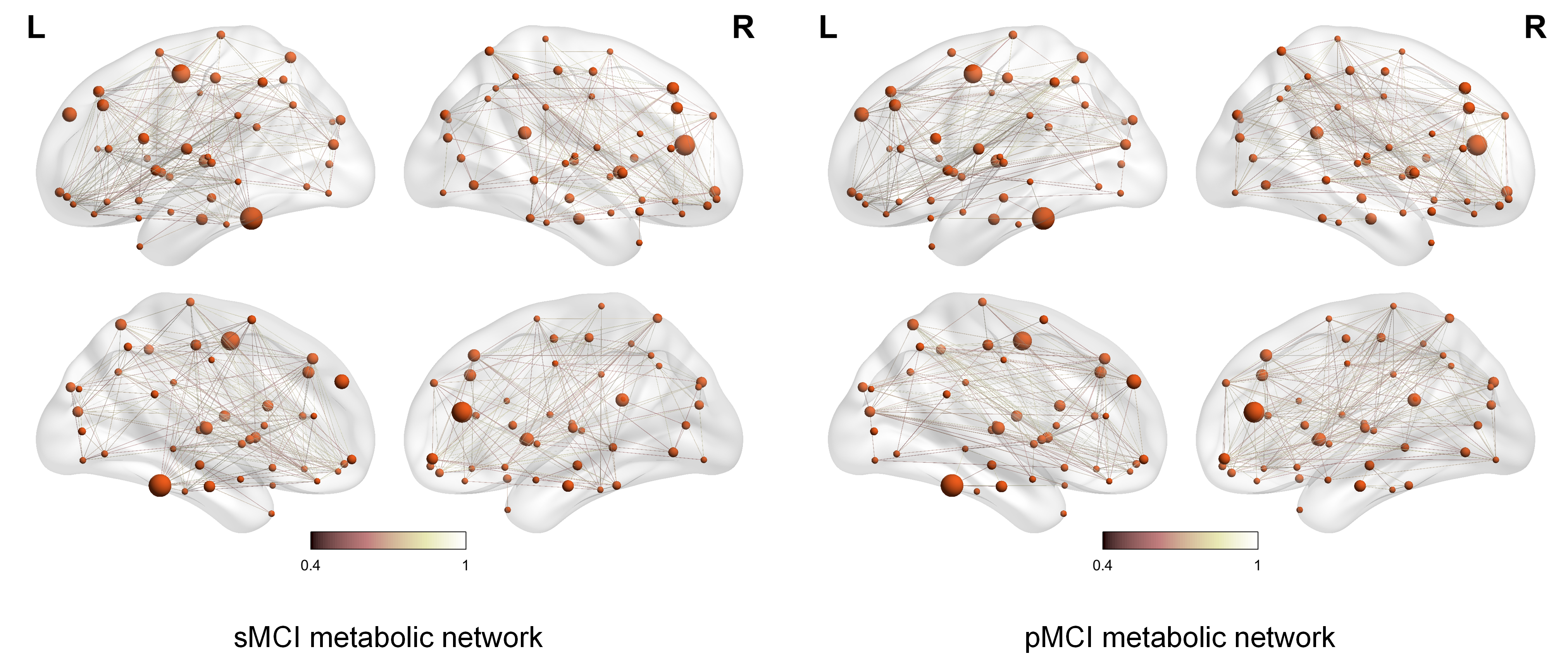


**Fig. A3** The metabolic brain network topography of a representative sMCI subject aged 72 years (MMSE: 30; APOE4: negative) and a pMCI subject aged 70 years (MMSE: 27; APOE4: positive). Connections linking pairs of nodes are represented by different hot colors. AAL regions are represented by spheres with radius proportional to the corresponding nodal degree


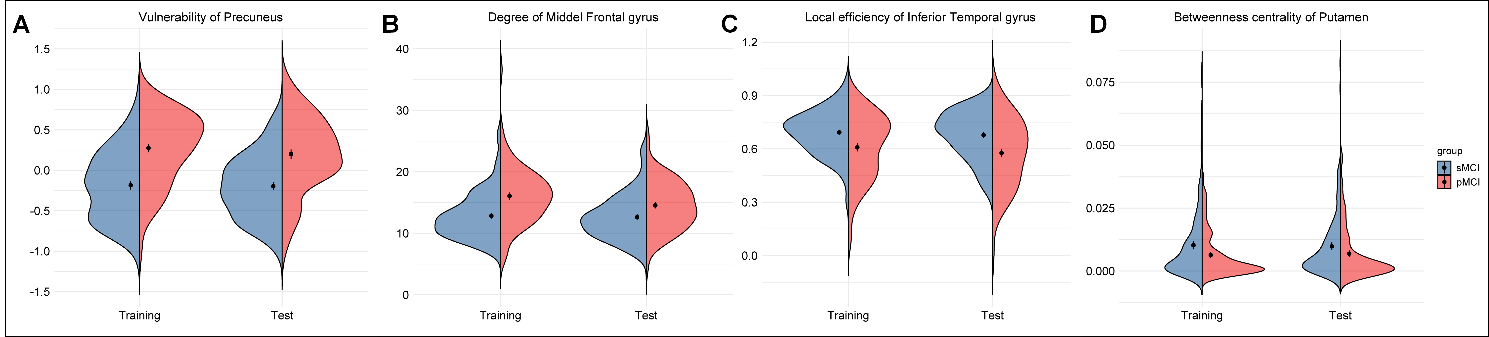


Fig. A4. The network properties of local brain region from sMCI and pMCI groups on training and test dataset. The violin plots were used to represent comparison of network properties distribution across different MCI groups and the black lines indicated MCI group mean and standard deviation.


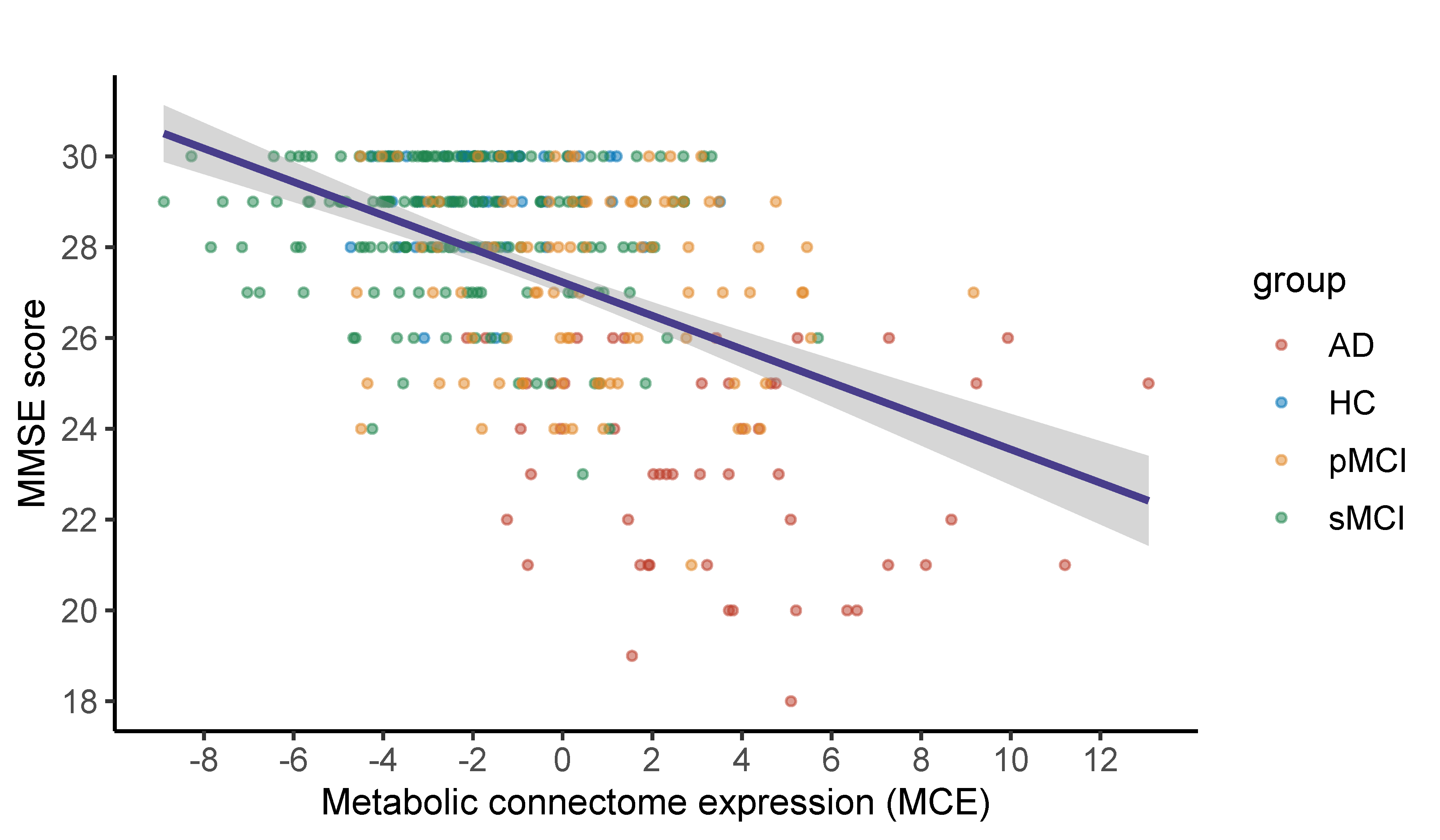


**Fig. A5** Correlation between metabolic connectome expression (MCE) and MMSE score (r = -0.483, *P* < 0.001; Pearson's product-moment correlation). The different color scatter points represent different diagnostic groups.


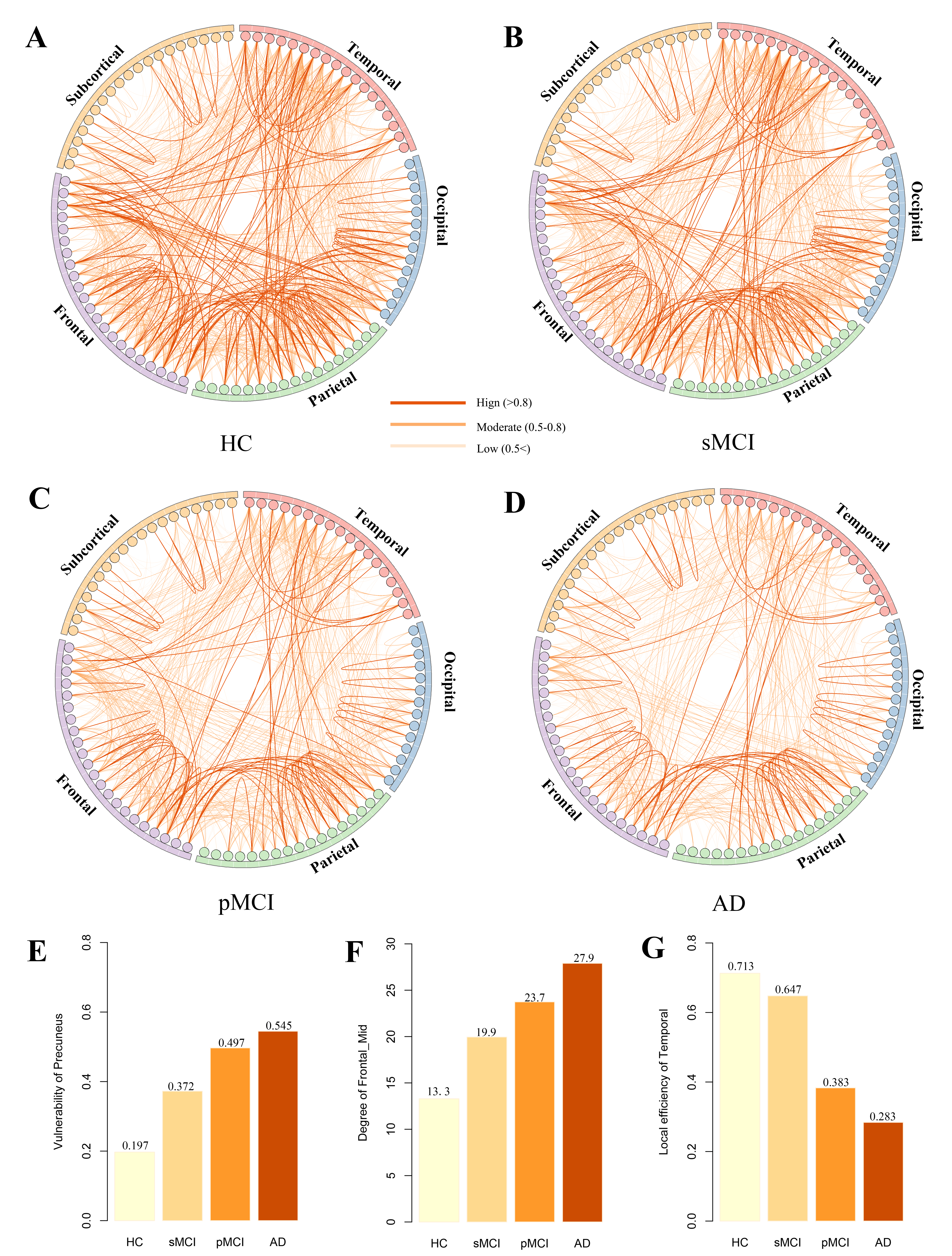


**Fig. A6** The metabolic connectome network topology in diagnostic groups: (A) healthy people, (B) sMCI group, (C) pMCI group and (D) AD group. The different colors of circle nodes indicated that they belonged to different anatomical brain regions (Pink: Temporal Lobe; Blue: Occipital Lobe; Yellow: Subcortical Nucleus; Green: Parietal Lobe; Purple: Frontal Lobe). The more red edges indicate more high connectivity between brain regions. Groups differences in connectome properties during AD progression: (E) Vulnerability, (F) Degree and (G) local efficiency

Reference

1. Botev ZI, Grotowski JF, Kroese DP. KERNEL DENSITY ESTIMATION VIA DIFFUSION. Ann Stat. 2010;38(5):2916-57. doi:10.1214/10-aos799.

2. Watts DJ, Strogatz SH. Collective dynamics of 'small-world' networks. Nature. 1998;393(6684):440-2. doi:10.1038/30918.

3. Latora V, Marchiori M. Efficient behavior of small-world networks. Phys Rev Lett. 2001;87(19):198701. doi:10.1103/PhysRevLett.87.198701.

4. Newman ME. Analysis of weighted networks. Physical review E, Statistical, nonlinear, and soft matter physics. 2004;70(5 Pt 2):056131. doi:10.1103/PhysRevE.70.056131.

5. Humphries MD, Gurney K. Network 'small-world-ness': a quantitative method for determining canonical network equivalence. PLoS One. 2008;3(4):e0002051. doi:10.1371/journal.pone.0002051.

6. Freeman LC. Centrality in social networks conceptual clarification. Social Networks. 1978;1(3):215-39. doi:<https://doi.org/10.1016/0378-8733(78)90021-7>.

7. Rubinov M, Sporns O. Complex network measures of brain connectivity: uses and interpretations. Neuroimage. 2010;52(3):1059-69. doi:10.1016/j.neuroimage.2009.10.003.
